# Inferring biological networks with output kernel trees

**DOI:** 10.1186/1471-2105-8-S2-S4

**Published:** 2007-05-03

**Authors:** Pierre Geurts, Nizar Touleimat, Marie Dutreix, Florence d'Alché-Buc

**Affiliations:** 1 IBISC FRE CNRS 2873 & Epigenomics project, GENOPOLE, 523, Place des Terrasses, 91 Evry, France; 2 Department of Electrical Engineering and Computer Science & GIGA, University of Liège, Institut Montefiore, Sart Tilman B28, 4000 Liège, Belgium; 3 UMR 2027 CNRS-IC, Institut Curie, Bâtiment 110, Centre Universitaire, 91405 Orsay, France

## Abstract

**Background:**

Elucidating biological networks between proteins appears nowadays as one of the most important challenges in systems biology. Computational approaches to this problem are important to complement high-throughput technologies and to help biologists in designing new experiments. In this work, we focus on the completion of a biological network from various sources of experimental data.

**Results:**

We propose a new machine learning approach for the supervised inference of biological networks, which is based on a kernelization of the output space of regression trees. It inherits several features of tree-based algorithms such as interpretability, robustness to irrelevant variables, and input scalability. We applied this method to the inference of two kinds of networks in the yeast *S. cerevisiae*: a protein-protein interaction network and an enzyme network. In both cases, we obtained results competitive with existing approaches. We also show that our method provides relevant insights on input data regarding their potential relationship with the existence of interactions. Furthermore, we confirm the biological validity of our predictions in the context of an analysis of gene expression data.

**Conclusion:**

Output kernel tree based methods provide an efficient tool for the inference of biological networks from experimental data. Their simplicity and interpretability should make them of great value for biologists.

## Background

The large spread-out of microarray data has recently renewed the interest for elucidating biological networks. Biological networks such as protein-protein interaction networks or metabolic networks are not real biological systems *per se *but are very convenient representations of the relations that underlie these complex systems. In this domain, the main challenge is to infer the structure of the networks from all available data for a given organism. Both supervised and unsupervised methods have been proposed to address this problem. Unsupervised methods derive some interaction score for each protein pair on the basis of single or multiple sources of data (e.g., [1]). The great advantage of these methods lies in the fact that they do not require any prior knowledge about the network structure. However, they potentially perform poorly in comparison with supervised methods that incorporate more information. Among supervised methods, mainly two approaches have been adopted. Relational learning approaches exploit a sample of known interacting and non-interacting protein pairs to learn a classifier that can decide if a new pair of proteins is interacting or not from a set of features defined directly on pairs [2]. Other supervised approaches adopt a more global view of the problem, searching to complete the protein network from a known subnetwork. These algorithms use features of a single protein (or gene) to determine the position of this protein in the network [3-5]. The work presented in this paper falls into this latter family of methods. Existing supervised algorithms often embed the input data used to infer the network in a kernel and thus result in black-box models that do not provide much insight about the problem. In this paper, we propose a new method, called Output Kernel Trees, based on a kernelization of the output space of regression trees. Unlike existing kernel-based methods, it uses the original (non kernelized) input space and thus fully inherits the interpretability and robustness to irrelevant variables of standard tree-based methods. When applied to network inference, it provides useful information about the relationship between the input data and the existence of interactions.

The paper is structured as follows. We first introduce the general setting of supervised inference of biological networks and show how this problem can be addressed using Output Kernel Trees. Numerical experiments concern two kinds of networks in the yeast *S. cerevisiae*: a protein-protein interaction network and an enzyme network. We compare and discuss the role of various input features from expression data to phylogenetic profiles for the prediction of interactions. Our algorithm obtains results competitive with existing approaches and offers a way to rank the features according to their importance in the prediction. We also illustrate the biological validity of our predictions in the context of an analysis of gene expression data.

## Methods

### Supervised network inference

The problem of supervised network inference has been defined in [3,6] and subsequently considered in [5]. It may be formulated as follows.

Let *G *= (*V*, *E*) be an undirected graph with vertices *V *and edges *E *⊂ *V *× *V*·|*V*| = *m *is the number of nodes in the graph. We suppose that each vertex *v*_*i *_, *i *= 1...*m*, can be described by some features in some input space X
 MathType@MTEF@5@5@+=feaafiart1ev1aaatCvAUfKttLearuWrP9MDH5MBPbIqV92AaeXatLxBI9gBamrtHrhAL1wy0L2yHvtyaeHbnfgDOvwBHrxAJfwnaebbnrfifHhDYfgasaacH8akY=wiFfYdH8Gipec8Eeeu0xXdbba9frFj0=OqFfea0dXdd9vqai=hGuQ8kuc9pgc9s8qqaq=dirpe0xb9q8qiLsFr0=vr0=vr0dc8meaabaqaciaacaGaaeqabaWaaeGaeaaakeaaimaacqWFxepwaaa@384F@, and we denote by *x*(*v*_*i*_) = *x*_*i *_∈ X
 MathType@MTEF@5@5@+=feaafiart1ev1aaatCvAUfKttLearuWrP9MDH5MBPbIqV92AaeXatLxBI9gBamrtHrhAL1wy0L2yHvtyaeHbnfgDOvwBHrxAJfwnaebbnrfifHhDYfgasaacH8akY=wiFfYdH8Gipec8Eeeu0xXdbba9frFj0=OqFfea0dXdd9vqai=hGuQ8kuc9pgc9s8qqaq=dirpe0xb9q8qiLsFr0=vr0=vr0dc8meaabaqaciaacaGaaeqabaWaaeGaeaaakeaaimaacqWFxepwaaa@384F@ this information. Only the knowledge of a subgraph *G*_*n *_= (*V*_*n*_, *E*_*n*_) of G is available during the training phase: without loosing generality, we enumerate the nodes belonging to *V*_*n *_as *v*_1_,....,*v*_*n *_where *n *is the number of nodes in the subgraph denoted by *G*_*n *_= (*V*_*n*_, *E*_*n*_) with *V*_*n *_⊂ *V *and *E*_*n *_= {(*v*, *v*') ∈ *E*|*v*, *v*' ∈ *V*_*n*_}. The goal of supervised graph inference is then to determine from the knowledge of *G*_*n *_a function *e*(*x*(*v*), *x*(*v*')): *V *× *V *→ {0,1}, ideally such that *e*(*x*(*v*), *x*(*v*')) = 1 ⇔ (*v*, *v*') ∈ *E*.

Following [3] and [5], our solution is based on a kernel embedding of the graph. A (positive semi-definite) kernel is defined as a function *k*: *V *× *V *→ ℛ
 MathType@MTEF@5@5@+=feaafiart1ev1aaatCvAUfKttLearuWrP9MDH5MBPbIqV92AaeXatLxBI9gBaebbnrfifHhDYfgasaacH8akY=wiFfYdH8Gipec8Eeeu0xXdbba9frFj0=OqFfea0dXdd9vqai=hGuQ8kuc9pgc9s8qqaq=dirpe0xb9q8qiLsFr0=vr0=vr0dc8meaabaqaciaacaGaaeqabaqabeGadaaakeaat0uy0HwzTfgDPnwy1egaryqtHrhAL1wy0L2yHvdaiqaacqWFBeIuaaa@377C@ which induces a feature map φ into a Hilbert space ℋ
 MathType@MTEF@5@5@+=feaafiart1ev1aaatCvAUfKttLearuWrP9MDH5MBPbIqV92AaeXatLxBI9gBamrtHrhAL1wy0L2yHvtyaeHbnfgDOvwBHrxAJfwnaebbnrfifHhDYfgasaacH8akY=wiFfYdH8Gipec8Eeeu0xXdbba9frFj0=OqFfea0dXdd9vqai=hGuQ8kuc9pgc9s8qqaq=dirpe0xb9q8qiLsFr0=vr0=vr0dc8meaabaqaciaacaGaaeqabaWaaeGaeaaakeaaimaacqWFlecsaaa@3763@ such that *k*(*v*, *v*') = ⟨ φ(*v*), φ((*v*')⟩. To solve the problem of graph inference, we first define a kernel *k*(*v*, *v*') such that adjacent vertices lead to high values of *k *and non-adjacent ones lead to smaller ones. The mapping of this kernel is thus such that φ(*v*) is close to φ(*v*') in ℋ
 MathType@MTEF@5@5@+=feaafiart1ev1aaatCvAUfKttLearuWrP9MDH5MBPbIqV92AaeXatLxBI9gBamrtHrhAL1wy0L2yHvtyaeHbnfgDOvwBHrxAJfwnaebbnrfifHhDYfgasaacH8akY=wiFfYdH8Gipec8Eeeu0xXdbba9frFj0=OqFfea0dXdd9vqai=hGuQ8kuc9pgc9s8qqaq=dirpe0xb9q8qiLsFr0=vr0=vr0dc8meaabaqaciaacaGaaeqabaWaaeGaeaaakeaaimaacqWFlecsaaa@3763@ as soon as *v *and *v' *are connected. Then, the problem of graph inference may be solved as follows: from the *n *× *n *Gram matrix *K *with *K*_*i*, *j *_= *k*(*v*_*i*_, *v*_*j*_) and the input feature vectors *x*_*i*_, find an approximation of the kernel values between pairs of new vertices described by their input values. A graph on unseen vertices is then obtained from the learnt kernel by connecting those vertices that correspond to a kernel prediction above some threshold.

A natural kernel between nodes of a graph is the diffusion kernel proposed in [7]. It defines the kernel value *k*(*v*_*i*_, *v*_*j*_) between nodes *v*_*i *_and *v*_*j *_as the (*i*, *j*)-element of the matrix *K *= *exp*(-*βL*), where *L *= *D *- *A *is the Laplacian matrix of the graph, with *D *the diagonal matrix of node connectivities and *A *the adjacency matrix, and *β *> 0 is a user-defined parameter that controls the degree of diffusion. With respect to the adjacency matrix, the diffusion kernel defines a more global and smoother similarity measure between two nodes that takes into account all paths in the graph (even non direct) between these two nodes. When *β *increases, the kernel diffuses more deeply into the graph, making distant vertices in the graph closer in ℋ
 MathType@MTEF@5@5@+=feaafiart1ev1aaatCvAUfKttLearuWrP9MDH5MBPbIqV92AaeXatLxBI9gBamrtHrhAL1wy0L2yHvtyaeHbnfgDOvwBHrxAJfwnaebbnrfifHhDYfgasaacH8akY=wiFfYdH8Gipec8Eeeu0xXdbba9frFj0=OqFfea0dXdd9vqai=hGuQ8kuc9pgc9s8qqaq=dirpe0xb9q8qiLsFr0=vr0=vr0dc8meaabaqaciaacaGaaeqabaWaaeGaeaaakeaaimaacqWFlecsaaa@3763@ with respect to directly adjacent vertices (see [7] for more details and several interpretations of the diffusion kernel).

### Output Kernel Trees

Output Kernel Trees (OK3, [8]) are a kernelization of standard classification and regression trees [9] that can handle any output space over which a kernel may be defined. By extension, this method also allows to learn a kernel as a function of an input vector. We focus our presentation here on this particular feature of the method. The interested reader may refer to [8] for a more complete description.

#### Learning stage

Our algorithm follows the main steps of the CART algorithm [9]. Starting from a training set of vertices {*v*_1_,...,*v*_*n*_} described by their input vectors *x*_*i *_= *x*(*v*_*i*_), *i *= 1,...,*n *and a Gram matrix *K *with *K*_*i*,*j *_= *k*(*v*_*i*_, *v*_*j*_), the idea of our method is to recursively split the training set with binary tests based on the input features. A test *T *is a boolean function of the input feature vector that usually involves only one feature at the same time: for a numerical variable, it compares its value to a threshold and for a categorical variable, it checks whether its value belongs to a subset of all possible values of the variable. Each split of a tree node aims at reducing as much as possible the variance of the output feature vector ϕ(*v*) in the left and right subsets of graph vertices corresponding to the two issues of the test. (Note that to avoid confusion between nodes of the output graph and nodes of the tree model, we reserve the term "vertex" for the former, and "node" for the latter.) Given the definition of the output kernel, this amounts at dividing the set of vertices corresponding to that node into two subsets in which vertices are as much as possible connected between each other in the training graph.

More precisely, the score used to evaluate and select a test *T *given the local learning sample *S *at the current node is defined as follows:

Score(T,S)=var⁡{φ(v)|S}−NlNvar⁡{φ(v)|Sl}−NrNvar⁡{φ(v)|Sr},     (1)
 MathType@MTEF@5@5@+=feaafiart1ev1aaatCvAUfKttLearuWrP9MDH5MBPbIqV92AaeXatLxBI9gBaebbnrfifHhDYfgasaacH8akY=wiFfYdH8Gipec8Eeeu0xXdbba9frFj0=OqFfea0dXdd9vqai=hGuQ8kuc9pgc9s8qqaq=dirpe0xb9q8qiLsFr0=vr0=vr0dc8meaabaqaciaacaGaaeqabaqabeGadaaakeaaieaacqWFtbWucqWFJbWycqWFVbWBcqWFYbGCcqWFLbqzcqGGOaakcqWGubavcqGGSaalcqWGtbWucqGGPaqkcqGH9aqpcyGG2bGDcqGGHbqycqGGYbGCcqGG7bWEiiGacqGFgpGzcqGGOaakcqWG2bGDcqGGPaqkcqGG8baFcqWGtbWucqGG9bqFcqGHsisldaWcaaqaaiabd6eaonaaBaaaleaacqWGSbaBaeqaaaGcbaGaemOta4eaaiGbcAha2jabcggaHjabckhaYjabcUha7jab+z8aMjabcIcaOiabdAha2jabcMcaPiabcYha8jabdofatnaaBaaaleaacqWGSbaBaeqaaOGaeiyFa0NaeyOeI0YaaSaaaeaacqWGobGtdaWgaaWcbaGaemOCaihabeaaaOqaaiabd6eaobaacyGG2bGDcqGGHbqycqGGYbGCcqGG7bWEcqGFgpGzcqGGOaakcqWG2bGDcqGGPaqkcqGG8baFcqWGtbWudaWgaaWcbaGaemOCaihabeaakiabc2ha9jabcYcaSiaaxMaacaWLjaWaaeWaaeaacqaIXaqmaiaawIcacaGLPaaaaaa@7708@

where *N *is the size of *S*, *S*_*l *_and *S*_*r *_are its left and right successors of size *N*_*l *_and *N*_*r *_respectively (corresponding to the test *T *being true or false respectively) and var{ϕ(*v*)|*S*} is the empirical variance of the output feature vector in the subset *S*, computed using the kernel trick by:

var⁡{φ(v)|S}=1N∑i=1N||φ(vi)−1N∑i=1Nφ(vi)||2=1N∑i=1Nk(vi,vi)−1N2∑i,j=1Nk(vi,vj).     (2)
 MathType@MTEF@5@5@+=feaafiart1ev1aaatCvAUfKttLearuWrP9MDH5MBPbIqV92AaeXatLxBI9gBaebbnrfifHhDYfgasaacH8akY=wiFfYdH8Gipec8Eeeu0xXdbba9frFj0=OqFfea0dXdd9vqai=hGuQ8kuc9pgc9s8qqaq=dirpe0xb9q8qiLsFr0=vr0=vr0dc8meaabaqaciaacaGaaeqabaqabeGadaaakeaacyGG2bGDcqGGHbqycqGGYbGCcqGG7bWEiiGacqWFgpGzcqGGOaaktCvAUfeBSjuyZL2yd9gzLbvyNv2CaeHbuLwBLnhiov2DGi1BTfMBaGabciaa+zhacqGGPaqkcqGG8baFcqWGtbWucqGG9bqFcqGH9aqpdaWcaaqaaiabigdaXaqaaiabd6eaobaadaaeWbqaaiabcYha8jabcYha8jab=z8aMjabcIcaOiabdAha2naaBaaaleaacqWGPbqAaeqaaOGaeiykaKIaeyOeI0caleaacqWGPbqAcqGH9aqpcqaIXaqmaeaacqWGobGta0GaeyyeIuoakmaalaaabaGaeGymaedabaGaemOta4eaamaaqahabaGae8NXdyMaeiikaGIaemODay3aaSbaaSqaaiabdMgaPbqabaGccqGGPaqkcqGG8baFcqGG8baFdaahaaWcbeqaaiabikdaYaaaaeaacqWGPbqAcqGH9aqpcqaIXaqmaeaacqWGobGta0GaeyyeIuoakiabg2da9maalaaabaGaeGymaedabaGaemOta4eaamaaqahabaGaem4AaSMaeiikaGIaemODay3aaSbaaSqaaiabdMgaPbqabaGccqGGSaalcqWG2bGDdaWgaaWcbaGaemyAaKgabeaakiabcMcaPaWcbaGaemyAaKMaeyypa0JaeGymaedabaGaemOta4eaniabggHiLdGccqGHsisldaWcaaqaaiabigdaXaqaaiabd6eaonaaCaaaleqabaGaeGOmaidaaaaakmaaqahabaGaem4AaSMaeiikaGIaemODay3aaSbaaSqaaiabdMgaPbqabaGccqGGSaalcqWG2bGDdaWgaaWcbaGaemOAaOgabeaakiabcMcaPiabc6caUaWcbaGaemyAaKMaeiilaWIaemOAaOMaeyypa0JaeGymaedabaGaemOta4eaniabggHiLdGccaWLjaGaaCzcamaabmaabaGaeGOmaidacaGLOaGaayzkaaaaaa@9F97@

Like in the standard CART algorithm, an exhaustive search is carried out at each tree node to find the test that maximizes this score. The splitting of a node is stopped when the output feature vector is constant in *S *(ie. variance (2) is null) or some stopping criterion is met (e.g., the size of the local subsample is below some threshold).

By analogy with regression trees, this algorithm actually tries to find implicitly an approximation φ^
 MathType@MTEF@5@5@+=feaafiart1ev1aaatCvAUfKttLearuWrP9MDH5MBPbIqV92AaeXatLxBI9gBaebbnrfifHhDYfgasaacH8akY=wiFfYdH8Gipec8Eeeu0xXdbba9frFj0=OqFfea0dXdd9vqai=hGuQ8kuc9pgc9s8qqaq=dirpe0xb9q8qiLsFr0=vr0=vr0dc8meaabaqaciaacaGaaeqabaqabeGadaaakeaaiiGacuWFgpGzgaqcaaaa@2E7C@(*x*(*v*)) of the output feature vector ϕ(*v*) corresponding to a vertex *v *from its input vector *x*(*v*). The loss function that it minimizes (in average) over the learning sample is the square distance in ℋ
 MathType@MTEF@5@5@+=feaafiart1ev1aaatCvAUfKttLearuWrP9MDH5MBPbIqV92AaeXatLxBI9gBamrtHrhAL1wy0L2yHvtyaeHbnfgDOvwBHrxAJfwnaebbnrfifHhDYfgasaacH8akY=wiFfYdH8Gipec8Eeeu0xXdbba9frFj0=OqFfea0dXdd9vqai=hGuQ8kuc9pgc9s8qqaq=dirpe0xb9q8qiLsFr0=vr0=vr0dc8meaabaqaciaacaGaaeqabaWaaeGaeaaakeaaimaacqWFlecsaaa@3763@, ie. ||φ^
 MathType@MTEF@5@5@+=feaafiart1ev1aaatCvAUfKttLearuWrP9MDH5MBPbIqV92AaeXatLxBI9gBaebbnrfifHhDYfgasaacH8akY=wiFfYdH8Gipec8Eeeu0xXdbba9frFj0=OqFfea0dXdd9vqai=hGuQ8kuc9pgc9s8qqaq=dirpe0xb9q8qiLsFr0=vr0=vr0dc8meaabaqaciaacaGaaeqabaqabeGadaaakeaaiiGacuWFgpGzgaqcaaaa@2E7C@(*x*(*v*)) - φ(*v*)||^2^.

#### Prediction stage

Again, by analogy with regression trees, each leaf *L *of the tree is labeled with a prediction φ^
 MathType@MTEF@5@5@+=feaafiart1ev1aaatCvAUfKttLearuWrP9MDH5MBPbIqV92AaeXatLxBI9gBaebbnrfifHhDYfgasaacH8akY=wiFfYdH8Gipec8Eeeu0xXdbba9frFj0=OqFfea0dXdd9vqai=hGuQ8kuc9pgc9s8qqaq=dirpe0xb9q8qiLsFr0=vr0=vr0dc8meaabaqaciaacaGaaeqabaqabeGadaaakeaaiiGacuWFgpGzgaqcaaaa@2E7C@_*L *_in ℋ
 MathType@MTEF@5@5@+=feaafiart1ev1aaatCvAUfKttLearuWrP9MDH5MBPbIqV92AaeXatLxBI9gBamrtHrhAL1wy0L2yHvtyaeHbnfgDOvwBHrxAJfwnaebbnrfifHhDYfgasaacH8akY=wiFfYdH8Gipec8Eeeu0xXdbba9frFj0=OqFfea0dXdd9vqai=hGuQ8kuc9pgc9s8qqaq=dirpe0xb9q8qiLsFr0=vr0=vr0dc8meaabaqaciaacaGaaeqabaWaaeGaeaaakeaaimaacqWFlecsaaa@3763@ computed as:

φ^L=1NL∑i=1NLφ(vi),     (3)
 MathType@MTEF@5@5@+=feaafiart1ev1aaatCvAUfKttLearuWrP9MDH5MBPbIqV92AaeXatLxBI9gBaebbnrfifHhDYfgasaacH8akY=wiFfYdH8Gipec8Eeeu0xXdbba9frFj0=OqFfea0dXdd9vqai=hGuQ8kuc9pgc9s8qqaq=dirpe0xb9q8qiLsFr0=vr0=vr0dc8meaabaqaciaacaGaaeqabaqabeGadaaakeaaiiGacuWFgpGzgaqcamaaBaaaleaacqWGmbataeqaaOGaeyypa0ZaaSaaaeaacqaIXaqmaeaacqWGobGtdaWgaaWcbaGaemitaWeabeaaaaGcdaaeWbqaaiab=z8aMjabcIcaOiabdAha2naaBaaaleaacqWGPbqAaeqaaOGaeiykaKIaeiilaWIaaCzcaiaaxMaadaqadaqaaiabiodaZaGaayjkaiaawMcaaaWcbaGaemyAaKMaeyypa0JaeGymaedabaGaemOta40aaSbaaWqaaiabdYeambqabaaaniabggHiLdaaaa@4768@

where *N*_*L *_is the number of learning cases that reach this leaf. Our final goal however is to make predictions about the kernel value between two vertices *v *and *v' *described by their input vectors *x*(*v*) and *x*(*v*'). Let us suppose that *x*(*v*) (resp. *x*(*v*')) reaches leaf *L*_1 _(resp. *L*_2_) that contains vertices {v11,...,vNL11}
 MathType@MTEF@5@5@+=feaafiart1ev1aaatCvAUfKttLearuWrP9MDH5MBPbIqV92AaeXatLxBI9gBaebbnrfifHhDYfgasaacH8akY=wiFfYdH8Gipec8Eeeu0xXdbba9frFj0=OqFfea0dXdd9vqai=hGuQ8kuc9pgc9s8qqaq=dirpe0xb9q8qiLsFr0=vr0=vr0dc8meaabaqaciaacaGaaeqabaqabeGadaaakeaacqGG7bWEcqWG2bGDdaqhaaWcbaGaeGymaedabaGaeGymaedaaOGaeiilaWIaeiOla4IaeiOla4IaeiOla4IaeiilaWIaemODay3aa0baaSqaaiabd6eaonaaBaaameaacqWGmbatdaWgaaqaaiabigdaXaqabaaabeaaaSqaaiabigdaXaaakiabc2ha9baa@3DCF@ (resp. {v12,...,vNL22}
 MathType@MTEF@5@5@+=feaafiart1ev1aaatCvAUfKttLearuWrP9MDH5MBPbIqV92AaeXatLxBI9gBaebbnrfifHhDYfgasaacH8akY=wiFfYdH8Gipec8Eeeu0xXdbba9frFj0=OqFfea0dXdd9vqai=hGuQ8kuc9pgc9s8qqaq=dirpe0xb9q8qiLsFr0=vr0=vr0dc8meaabaqaciaacaGaaeqabaqabeGadaaakeaacqGG7bWEcqWG2bGDdaqhaaWcbaGaeGymaedabaGaeGOmaidaaOGaeiilaWIaeiOla4IaeiOla4IaeiOla4IaeiilaWIaemODay3aa0baaSqaaiabd6eaonaaBaaameaacqWGmbatdaWgaaqaaiabikdaYaqabaaabeaaaSqaaiabikdaYaaakiabc2ha9baa@3DD5@). From (3), the kernel between *v *and *v' *is approximated by:

k^(v,v′)=〈φ^L1,φ^L2〉=1NL1NL2∑i=1NL1∑j=1NL2〈φ(vi1),φ(vj2)〉=1NL1NL2∑i=1NL1∑j=1NL2k(vi1,vj2),     (4)
 MathType@MTEF@5@5@+=feaafiart1ev1aaatCvAUfKttLearuWrP9MDH5MBPbIqV92AaeXatLxBI9gBaebbnrfifHhDYfgasaacH8akY=wiFfYdH8Gipec8Eeeu0xXdbba9frFj0=OqFfea0dXdd9vqai=hGuQ8kuc9pgc9s8qqaq=dirpe0xb9q8qiLsFr0=vr0=vr0dc8meaabaqaciaacaGaaeqabaqabeGadaaakeaaieGacuWFRbWAgaqcaiabcIcaOiabdAha2jabcYcaSiqbdAha2zaafaGaeiykaKIaeyypa0JaeyykJeocciGaf4NXdyMbaKaadaWgaaWcbaGaemitaW0aaSbaaWqaaiabigdaXaqabaaaleqaaOGaeiilaWIaf4NXdyMbaKaadaWgaaWcbaGaemitaW0aaSbaaWqaaiabikdaYaqabaaaleqaaOGaeyOkJeVaeyypa0ZaaSaaaeaacqaIXaqmaeaacqWGobGtdaWgaaWcbaGaemitaW0aaSbaaWqaaiabigdaXaqabaaaleqaaOGaemOta40aaSbaaSqaaiabdYeamnaaBaaameaacqaIYaGmaeqaaaWcbeaaaaGcdaaeWbqaamaaqahabaGaeyykJeUae4NXdyMaeiikaGIaemODay3aa0baaSqaaiabdMgaPbqaaiabigdaXaaakiabcMcaPiabcYcaSiab+z8aMjabcIcaOiabdAha2naaDaaaleaacqWGQbGAaeaacqaIYaGmaaGccqGGPaqkcqGHQms8cqGH9aqpdaWcaaqaaiabigdaXaqaaiabd6eaonaaBaaaleaacqWGmbatdaWgaaadbaGaeGymaedabeaaaSqabaGccqWGobGtdaWgaaWcbaGaemitaW0aaSbaaWqaaiabikdaYaqabaaaleqaaaaakmaaqahabaWaaabCaeaacqWGRbWAcqGGOaakcqWG2bGDdaqhaaWcbaGaemyAaKgabaGaeGymaedaaOGaeiilaWIaemODay3aa0baaSqaaiabdQgaQbqaaiabikdaYaaakiabcMcaPiabcYcaSiaaxMaacaWLjaWaaeWaaeaacqaI0aanaiaawIcacaGLPaaaaSqaaiabdQgaQjabg2da9iabigdaXaqaaiabd6eaonaaBaaameaacqWGmbatdaWgaaqaaiabikdaYaqabaaabeaaa0GaeyyeIuoaaSqaaiabdMgaPjabg2da9iabigdaXaqaaiabd6eaonaaBaaameaacqWGmbatdaWgaaqaaiabigdaXaqabaaabeaaa0GaeyyeIuoaaSqaaiabdQgaQjabg2da9iabigdaXaqaaiabd6eaonaaBaaameaacqWGmbatdaWgaaqaaiabikdaYaqabaaabeaaa0GaeyyeIuoaaSqaaiabdMgaPjabg2da9iabigdaXaqaaiabd6eaonaaBaaameaacqWGmbatdaWgaaqaaiabigdaXaqabaaabeaaa0GaeyyeIuoaaaa@9C4E@

which makes use of kernel values only. Then, this kernel can be thresholded to make a prediction about the existence of an edge between *v *and *v'*.

By construction, our method, called Output Kernel Trees (OK3), shares several features of standard tree-based methods. The most attractive ones being the interpretability of the model and the ability of the method to rank the features (see below).

#### Ensembles of output kernelized trees

While useful for interpretability reasons, single trees are usually not competitive with other methods in terms of accuracy, essentially because of their high variance. Thus, in the context of classification and regression problems, ensemble methods have been proposed to reduce variance and improve accuracy. In general, these methods grow an ensemble of diverse trees instead of a single one and then combine in some fashion the predictions of these trees to yield a final prediction. Among these methods, those which only rely on score computations to grow the ensemble of trees and which combine predictions by simply averaging them, can be directly extended to OK3. As a matter of fact, the prediction of an ensemble of trees in ℋ
 MathType@MTEF@5@5@+=feaafiart1ev1aaatCvAUfKttLearuWrP9MDH5MBPbIqV92AaeXatLxBI9gBamrtHrhAL1wy0L2yHvtyaeHbnfgDOvwBHrxAJfwnaebbnrfifHhDYfgasaacH8akY=wiFfYdH8Gipec8Eeeu0xXdbba9frFj0=OqFfea0dXdd9vqai=hGuQ8kuc9pgc9s8qqaq=dirpe0xb9q8qiLsFr0=vr0=vr0dc8meaabaqaciaacaGaaeqabaWaaeGaeaaakeaaimaacqWFlecsaaa@3763@, which is an average of sums like (3), may be written as a weighted sum of output feature space vectors from the learning sample, i.e. φ^ens(x(v))=∑i=1nwi(v)φ(vi)
 MathType@MTEF@5@5@+=feaafiart1ev1aaatCvAUfKttLearuWrP9MDH5MBPbIqV92AaeXatLxBI9gBaebbnrfifHhDYfgasaacH8akY=wiFfYdH8Gipec8Eeeu0xXdbba9frFj0=OqFfea0dXdd9vqai=hGuQ8kuc9pgc9s8qqaq=dirpe0xb9q8qiLsFr0=vr0=vr0dc8meaabaqaciaacaGaaeqabaqabeGadaaakeaaiiGacuWFgpGzgaqcamaaBaaaleaacqWGLbqzcqWGUbGBcqWGZbWCaeqaaOGaeiikaGIaemiEaGNaeiikaGIaemODayNaeiykaKIaeiykaKIaeyypa0ZaaabmaeaacqWG3bWDdaWgaaWcbaGaemyAaKgabeaakiabcIcaOiabdAha2jabcMcaPaWcbaGaemyAaKMaeyypa0JaeGymaedabaGaemOBa4ganiabggHiLdGccqWFgpGzcqGGOaakcqWG2bGDdaWgaaWcbaGaemyAaKgabeaakiabcMcaPaaa@4D8E@. Then, kernel predictions are computed from the ensemble by k^ens(v,v′)=∑i=1n∑j=1nwi(v)wj(v′)k(vi,vj)
 MathType@MTEF@5@5@+=feaafiart1ev1aaatCvAUfKttLearuWrP9MDH5MBPbIqV92AaeXatLxBI9gBaebbnrfifHhDYfgasaacH8akY=wiFfYdH8Gipec8Eeeu0xXdbba9frFj0=OqFfea0dXdd9vqai=hGuQ8kuc9pgc9s8qqaq=dirpe0xb9q8qiLsFr0=vr0=vr0dc8meaabaqaciaacaGaaeqabaqabeGadaaakeaaieGacuWFRbWAgaqcamaaBaaaleaacqWGLbqzcqWGUbGBcqWGZbWCaeqaaOGaeiikaGIaemODayNaeiilaWIafmODayNbauaacqGGPaqkcqGH9aqpdaaeWaqaamaaqadabaGaem4DaC3aaSbaaSqaaiabdMgaPbqabaGccqGGOaakcqWG2bGDcqGGPaqkcqWG3bWDdaWgaaWcbaGaemOAaOgabeaakiabcIcaOiqbdAha2zaafaGaeiykaKIaem4AaSMaeiikaGIaemODay3aaSbaaSqaaiabdMgaPbqabaGccqGGSaalcqWG2bGDdaWgaaWcbaGaemOAaOgabeaakiabcMcaPaWcbaGaemOAaOMaeyypa0JaeGymaedabaGaemOBa4ganiabggHiLdaaleaacqWGPbqAcqGH9aqpcqaIXaqmaeaacqWGUbGBa0GaeyyeIuoaaaa@5CEA@. In our experiments, we grow ensembles of OK3 with the extra-trees method proposed in [10]. In this method, each tree of the ensemble is grown from the complete learning sample while randomizing the choice of the split at each node. We refer the interested reader to [10] for the exact description of this algorithm.

#### Attribute selection and ranking

An important feature of tree-based methods is that they can be exploited to rank attributes according to their importance for predicting the output value. The computation of this ranking is especially interesting with ensemble methods which are not interpretable by themselves. In the context of OK3, we propose to compute the importance of an attribute by computing for each split (in a tree, or in an ensemble of trees) where the attribute is used the total reduction of variance brought by the split, which is actually *N *× Score(*S*, *T*) (see Eqn. 1), and by summing these reductions. Thus, attributes that do not appear in a tree have an importance of zero, and those that are selected close to the root nodes of the trees typically receive high scores.

## Results and discussion

### Data

#### Biological networks

We carry out experiments on two kinds of protein networks in the yeast *S. cerevisiae*. The first one is a network of physical protein-protein interactions borrowed from [5] that consists of the high confidence interactions highlighted in [11]. It is composed of 2438 interactions that link 984 proteins. The second network is a network related to the metabolism of the yeast. Two proteins (enzymes) in this network are connected if they catalyze successive reactions in any metabolic pathway. It was obtained from the KEGG/PATHWAY database [12] by [4] and contains 668 proteins and 2782 edges. (Note that this network is slightly different from the one used in [3] and subsequently in [5].) 184 proteins are shared between the protein interaction network and the metabolic network.

As described in the Methods section, both networks were smoothed by a diffusion kernel. For comparison purpose with [5] and [4], the kernel matrix was normalized and the parameter *β *of the diffusion kernel was fixed to 3.0 for the protein-protein interaction network and to 1.0 for the metabolic network. We have nevertheless tried different values of *β *∈ [0.0, 3.0] but did not notice any important change in accuracy.

#### Input features

Different sources of data could be used for the inference of these biological networks. Experimental data obtained from various large scale methods are natural candidates but other kinds of data such as GO or KEGG annotations have also been used for this task [2]. In this paper, we used the same kinds of data as in [3] and [5].

#### Expression data (expr)

We considered two sets of gene expression data. The first dataset comes from the study in [13] and the second one comes from [14]. Both datasets contain small expression time series related to the cell-cycle in the yeast. Spellman et al's data gathers 77 time points and Eisen et al's data 80 time points. In our experiments, we use the original datasets accompanying the two publications, only filling missing values by the median of the corresponding column. Subsequently, we will refer to this data as "expr".

#### Phylogenetic profiles (phy)

The existence of orthologs of a given gene in a set of species is potentially an important source of information for the prediction of biological networks. In our experiments, we use the phylogenetic profiles gathered by [4]. They were obtained from the orthologous clusters in KEGG. Only fully sequenced genomes are taken into account. Each protein is described by a vector of 145 binary values, each one coding for the presence or the absence of an orthologous protein in a given organism.

#### Localization data (loc)

The localization of a protein in the cell is also potentially influencing its interactions with other proteins. The vector of features in this case consists of 23 binary values coding for the presence/absence of the protein in a given intracellular location. This data was obtained from the experiment in [15].

#### Yeast two hybrid network (y2h)

Such data is considered as a very noisy version of the true protein-protein interaction network and has been shown to contain many false positives. In our experiments, we use the networks obtained from the assays in [16] and [17].

Because of its pairwise nature, this kind of data can not be directly handled by tree-based methods that require that all proteins are described by an input feature vector. To still accommodate with it, we use the following procedure: following [3], we construct a graph with an edge between two proteins if these two proteins are connected in at least one of the two networks ([16] or [17]) and turn this graph into a kernel matrix using a diffusion kernel with *β *= 1.0. This kernel is then transformed into a input feature vector for each protein by computing the first 50 directions with kernel PCA.

### Results

For both networks, we use an ensemble of 100 output kernel trees grown with the extra-trees method with default parameters. To match the protocols used in [4] and [5], we evaluate the method by ten-fold cross-validation. On each run, we compute the diffusion kernel on 9 folds, apply OK3 and then compute from the resulting model all kernel predictions that involve at least one protein from the test fold. A network can then be reconstructed by connecting protein pairs with a kernel value above a threshold.

#### ROC analysis

We analyze ROC curves obtained by varying the threshold, the true positive rate being the proportion of existing edges correctly predicted and the false positive rate the proportion of non existing edges erroneously predicted. We (vertically) average the ROC curves obtained on the different folds and we also compute (average) areas under the ROC curves (AUC values).

**Figure 1:**
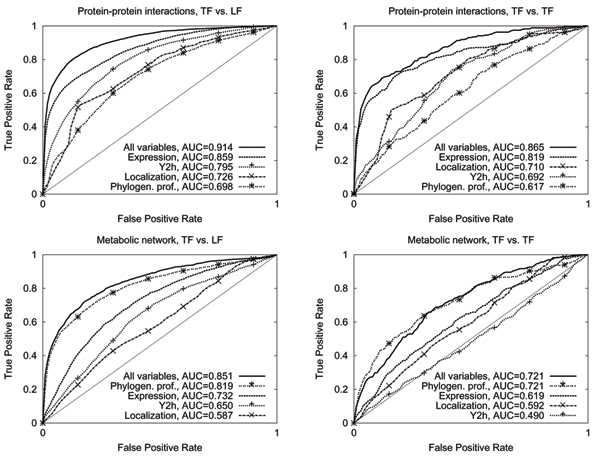
**ROC curves**. ROC curves for TF vs. LF edges (left) and TF vs. TF edges (right) with different sets of inputs, on the protein-protein interaction network (top) and the metabolic network (bottom).

We distinguish two types of edges for the computation of ROC curves: edges connecting an unseen protein (from the test fold, TF) to a seen protein (from the learning folds, LF) and edges connecting an unseen protein to another unseen protein (TF vs TF). We expect that the latter will be more difficult to predict than the former. Hence, we compute in each experiment three ROC curves and AUC values: the ROC curve computed on TF vs TF edges, on TF vs LF edges, and on both kinds of edges simultaneously. The TF vs. LF and TF vs. TF ROC curves with different sets of variable are given in Figure 1 for both networks. Average and standard errors of the AUC values are summarized in Table 1.

Overall, the results are quite good. They are better for the protein-protein interaction network than for the metabolic network. The way the method exploits each data source is very different in both networks. For the protein-protein interaction network, the most important source of information is the expression data followed by the y2h network, localization data, and phylogenetic profiles. For the prediction of the metabolic network, the most important source of information is the phylogenetic profiles followed by the expression data. Localization and y2h data are on the other hand not very useful on this latter database. On both networks, combining all data sources allows to improve the AUC values with respect to the use of each data source separately. As expected, TF vs. LF edges are easier to predict than TF vs. TF edges. The difference between the two kinds of edges is however less important on the protein-protein network than on the metabolic network.

This difference in AUC between the two networks probably reflects the biological significance of the input data. Actually, localization and y2h data directly reflect protein-protein interactions. In contrast, though interacting proteins belong per se to a same metabolic pathway, the inverse is not true. Indeed, non interacting proteins can participate to distant steps of a same pathway. In that case the localization and y2h network data would poorly contribute to prediction. Phylogenetic profiles are related to protein-protein interactions as well as pathway distribution since one expects all enzymes of the same pathway to be conserved or lost during evolution. The order of the different data set contributions to prediction nicely reflects all these biological constraints. Interestingly, expression data appear to be a good predictor for protein-protein interactions. This result could reflect the requirement that different partners of a protein complex should be co-expressed.

#### Comparison with full kernel-based methods

For comparison, the last column of Table 1 reports the results obtained in [5] for the protein-protein interaction network and in [4] for the metabolic network (when available). In both cases, the protocols are rigorously identical to ours, although the random folds of cross-validation are different. Both methods exploit a kernel on the inputs. [5] uses an algorithm based on expectation-maximization to learn simultaneously the missing kernel values and a weight for each different data source. [4] compares two approaches: kernel canonical correlation analysis and a distance metric learning method [6]. Several other approaches (such as a number of unsupervised methods) are also compared in these papers. We only report here their best results.

Looking at the AUC obtained when integrating all data sources (except y2h for the metabolic network that was not used in [4]), we get slightly worse results than the methods in [5] for the protein-protein interaction network and better results than the methods in [4] for the metabolic network. Note however that [5] reports an AUC of 0.858 for the prediction of TF vs. TF edges, which is slightly worse than our method (0.865). There are important differences with these methods in the exploitation of the individual data sources. On the protein-protein data, we are doing a much better use of the expression data and the y2h network while these methods are better in exploiting localization data and phylogenetic profiles. The results with y2h data is quite surprising since such kind of graph structured data seems at first more naturally handled by kernel-based methods. On the metabolic network however, we make a much better use of phylogenetic profiles than kernel-based methods and handle localization and expression data equivalently.

Kernel-based methods are usually not as efficient as tree-based ensemble methods to detect irrelevant inputs (although there exist techniques to incorporate specific attribute selection constraints into kernel-based methods). This may explain why they are not as good as our tree-based ensemble method on the expression data, which potentially contain irrelevant and noisy information. On the other hand, tests on phylogenetic variables in our trees are based on the presence or the absence of an ortholog protein in only one organism at a time. For the prediction of protein-protein interactions, the whole profile should be considered and hence these very local tests are somewhat inappropriate. For the prediction of the metabolic network, however, it is known that different organisms have developed different pathways.

Hence, the presence of an ortholog of the protein in a given organism is potentially informative. This may explain why our trees make a better use of phylogenetic profiles for the metabolic network than for the protein-protein network, while the opposite is true for kernel-based methods.

### Interpretability

One of the main advantages of our tree-based approach is that it provides interpretable results. We illustrate this feature in this section.

#### Clustering

**Figure 2:**
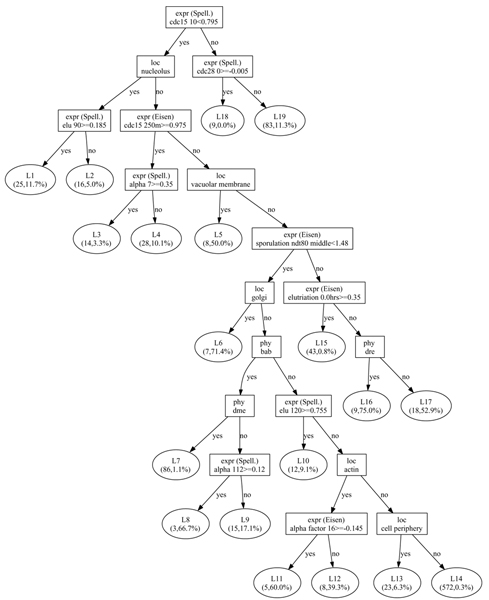
**Decision tree**. A decision tree obtained on the protein-protein interaction network using expression data, phylogenetic profiles and localization data as inputs. The tree size was determined by cost-complexity pruning with 10-fold cross-validation. The left (resp. right) edge from a test node corresponds to the test of the node being true (resp. false). Each leaf is labeled with a pair *(N*, *p*), where *N *is the number of proteins in its cluster and *p *is the percentage of protein pairs that interact in the cluster.

**Figure 3:**
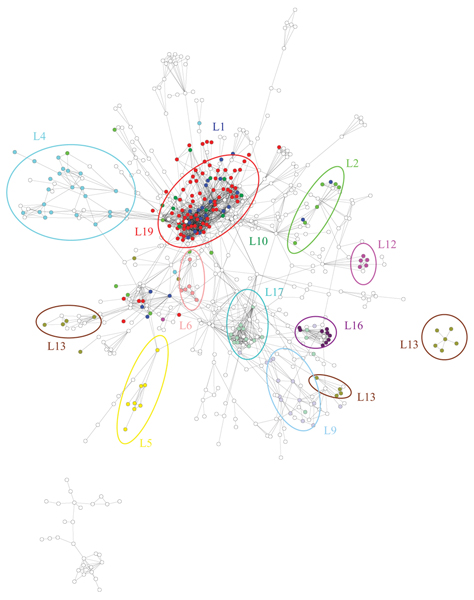
**Graph clustering**. The projection of the tree leaves in Figure 2 on the protein-protein interaction network. Only the leaves that contain more than 5 proteins and 5% of connections are represented.

When used as single trees, output kernel trees provide a partition of the learning sample into clusters, one for each tree leaf, where proteins are as much as possible connected between each other. Each cluster is furthermore described by a rule based on the input variables. As an illustration, Figure 2 shows a tree that was obtained from the whole learning sample on the protein-protein interaction data, using phylogenetic profiles, expression, and localization data as inputs. The tree complexity was automatically adjusted by cost-complexity pruning with 10-fold cross-validation [9]. The left (resp. right) successor of each test node corresponds to the test at the node being true (resp. false). Each leaf is labeled with a pair *(N*, *p*), where *N *is the number of proteins in its cluster and *p *is the percentage of protein pairs that interact in the cluster. For comparison, the percentage of interactions in the whole learning sample is 0.5%. Of course, since the problem is quite difficult and noisy, several leaves do not correspond to significantly connected proteins. We projected the more significant leaves (arbitrarily defined as those that contain more than 5 proteins and 5% of connections) on the protein-protein interaction network (see Figure 3). As expected, these clusters correspond to highly connected regions in the graph. Looking at tree tests, we get furthermore a description of these clusters in terms of the input features. For example, the leaf L19 corresponds to those genes that satisfy two conditions on experiments CDC15 and CDC28 of Spellman et al's expression data. They are represented by red nodes in the graph of Figure 3. An analysis of the GO functions of these genes shows that most of them participate to ribosome biogenesis.

#### Variable ranking

Table 2 shows the first 10 variables in the ranking obtained from the two datasets by ensembles of output kernel trees with expressions, phylogenetic profiles, and localization data. These rankings were obtained from ensembles of extra-trees with the importance measure developed in the Methods section. To further reduce the variance of these rankings, the importance of each feature is actually the average of the importances obtained over the 10 folds of the cross-validation.

Note that these rankings of individual features refine the ranking of the different data sources that was found in Table 1.

### Biological validation

**Figure 4:**
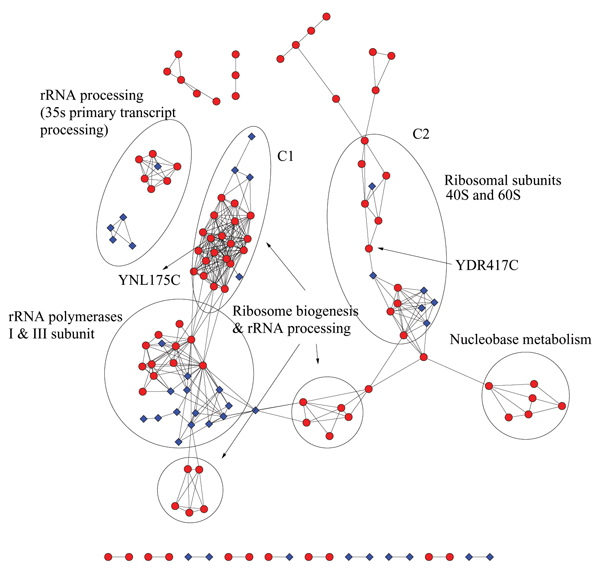
**Cluster prediction**. Predictions of protein-protein interactions in a cluster of 198 genes. Blue diamond-shaped nodes are proteins present in the training sample, red circle-shaped nodes were not seen by the learning algorithm. Annotation was found using BiNGO.

Previous experiments show the good general behavior of our algorithm on two benchmark problems. However, for this algorithm to be useful for biologists, it must be able to provide new and biologically sound predictions. To illustrate this capability, we run an additional experiment in the context of a bioinformatics analysis of a gene expression dataset. This transcriptome dataset, described in [18], includes gene expression kinetics of seven yeast strains submitted to a stress of radiation. A clustering analysis applied on these gene expression kinetics revealed several clusters of co-expressed genes, among which one cluster of 198 genes was deemed of particular interest for further analysis (see [19]). In this illustration, we focus on the prediction of protein-protein interactions in this cluster. As a training set for our algorithm, we use a recent interactome dataset proposed in [20]. This high-quality data set was obtained by intersecting data generated by several different interaction detection methods. The resulting network, called "filtered yeast interactome" (FYI), contains 1,379 proteins and 2,493 interactions. Only 60 among the 198 proteins in the cluster of interest are present in the FYI dataset, leaving the connections between the remaining proteins unknown. We learned a model from the FYI data using as inputs expression, localization, and phylogenetic data (the y2h data was not considered as it was one of the sources exploited to make up the FYI dataset) and then used this model to complete the network of interactions for the 198 genes. This resulted in a network with 379 edges (using a kernel threshold of 0.85), among which only 35 edges were previously known. Figure 4 draws this network where nodes present in the training set are represented by blue diamond-shaped nodes and unseen nodes by red circle-shaped nodes. Only proteins that are connected to at least one other protein are represented (in total, 131 out of 198). The network with all protein names is available in a web appendix [21].

First, we note that this cluster of co-regulated genes is highly connected. Indeed, using the same kernel threshold, a set of 198 random genes would contain in average 10 times less edges than our clu networks.ster. The inferred network thus suggests that these proteins are likely to share some functions. It also clearly reveals several highly connected subclusters of nodes that could correspond to several functional modules. To check this hypothesis, we use the gene ontology to annotate the different subnetworks in Cytoscape [22]. Statistical significance of the annotation was checked with BiNGO [23].

Figure 4 shows these annotations. This analysis highlights four distinct but related biological processes, all involved in different steps of the production of ribosomes. Interestingly, rRNA polymerases subunits and ribosomal subunits have no direct connections between themselves but both are connected with proteins involved in rRNA processing and ribosome biogenesis, which translates some biological facts. We thus retrieve with our method biologically meaningful subnetworks. Note that these subclusters are also highlighted in [19] by exploiting other sources of information (a.o., functional annotations, protein complexes, regulation related descriptors).

A finer way to validate our method is to try to infer the functions of unannotated proteins by looking at functions of their direct neighbors in the inferred network. As an illustration of this possibility, we first focused on the highly connected subnetwork C1. This subcluster contains six proteins, YNL175C, YCR016W, YDR365C, YKR060W, YBL028C and YOR206W, that were not yet annotated in our version of the annotation (dating of June 2006). However, their positions in the inferred network suggest that they participate in ribosome biogenesis. For some of them, it is indeed possible to find some clues that they are related to this process. For example, YNL175C shares some sequence similarity with YOL041C, which is itself involved in ribosome biogenesis. As a strong evidence in favor of our prediction, we note that a recent computational analysis [24] based on gene expression data and sequence analysis has concluded that all these six proteins participate in ribosome biogenesis. As a matter of fact, the GO annotation of these genes has been introduced in the Saccharomyces Genome Database [25] in September 2006. Another interesting protein is YDR417C whose function is yet unknown but which lies in the middle of a subset of proteins that are all components of the ribosomal subunits (subcluster C2 in Figure 4). Actually, it turns out that this protein has a large overlap in terms of DNA sequence with another protein, YDR418W, which is a component of the large ribosomal subunit. Its position in the network may thus come from the fact that probes on microarray may not specifically distinguish between two messengers coded by the same chromosome sequence.

## Conclusion

We proposed a new method for the supervised inference of biological networks. This method is based on a kernelization of the output space of tree-based methods. It yields competitive results with respect to full kernel-based methods on a protein-protein interaction network and on an enzyme network. In addition, it provides interpretable results in the form of a rule based clustering of the network and a ranking of the variables according to their importance at predicting new edges. The ability to discover new information about unannotated proteins was further illustrated on a small-scale study. These results suggest that our tool could be helpful to point out proteins that are worth further experimental investigations.

## Authors' contributions

PG developed the algorithm, carried out the experiments, and drafted the manuscript. FAB coordinated the collaboration, participated in the design of the algorithm, and helped in writing the manuscript. NT and MD helped in collecting and interpreting the data and did the biological validation of the results. All authors read and approved the final manuscript.

## Figures and Tables

**Table 1:** AUC results.

Inputs	All	TF vs. LF	TF vs. TF	Kern. (All)
Protein-protein interactions				

expr	0.851 ± 0.028	0.859 ± 0.027	0.819 ± 0.082	0.776
phy	0.693 ± 0.036	0.698 ± 0.035	0.617 ± 0.064	0.767
loc	0.725 ± 0.018	0.726 ± 0.017	0.710 ± 0.055	0.788
expr+phy+loc	0 887 ± 0 024	0 891 ± 0 023	0 845 ± 0 081	-
y2h	0.790 ± 0.023	0.795 ± 0.022	0.692 ± 0.068	0.612
expr+phy+loc+y2h	0.910 ± 0.019	0.914 ± 0.017	0.865 ± 0.057	0.939

Metabolic network				

expr	0.714 ± 0.032	0.732 ± 0.035	0.619 ± 0.089	0.706
Phy	0.815 ± 0.033	0.819 ± 0.031	0.721 ± 0.086	0.747
loc	0.587 ± 0.022	0.587 ± 0.022	0.592 ± 0.042	0.577
expr+phy+loc	0.847 ± 0.025	0.853 ± 0.025	0.733 ± 0.057	0.804
y2h	0.639 ± 0.033	0.650 ± 0.034	0.490 ± 0.098	-
expr+phy+loc+y2h	0.844 ± 0.025	0.851 ± 0.026	0.721 ± 0.056	-

AUC results obtained with extra-trees and ten-fold cross-validation compared with full kernel-based methods. The best result in each row between tree-based and kernel-based methods (for all predictions) is underlined.

**Table 2:** Variable ranking.

Protein-protein interactions			Metabolic network		
#	Att.	Imp	#	Att.	Imp

1	loc – nucleolus	0.021	1	phy – dre	0.011
2	expr (Spell.) – elu 120	0.013	2	phy – rno	0.009
3	loc – cytoplasm	0.012	3	expr (Eisen) – cdc15 120 m	0.008
4	expr (Eisen) – sporulation ndt80 early	0.012	4	phy – ecu	0.008
5	loc – nucleus	0.012	5	expr (Eisen) – cdc15 160 m	0.008
6	expr (Eisen) – sporulation 30 m	0.011	6	phy – pfa	0.007
7	expr (Eisen) – sporulation ndt80 middle	0.010	7	phy – mmu	0.007
8	expr (Spell.) – alpha 14	0.010	8	loc – cytoplasm	0.006
9	expr (Spell.) – elu 150	0.010	9	expr (Eisen) – cdc15 30 m	0.005
10	loc – mitochondrion	0.009	10	expr (Eisen) – elutriation 5.5 hrs	0.005

Variable rankings obtained with expressions, phylogenetic profiles, and localization data used as inputs to extra-trees.
